# Association between the modified Nutrition Risk in Critically Ill (mNUTRIC) score and clinical outcomes in the intensive care unit: a secondary analysis of a large prospective observational study

**DOI:** 10.1186/s12871-021-01439-x

**Published:** 2021-09-08

**Authors:** Na Wang, Mei-Ping Wang, Li Jiang, Bin Du, Bo Zhu, Xiu-Ming Xi

**Affiliations:** 1 Emergency Department of China Rehabilitation Research Center, Fengtai District, Capital Medical University, no.10 Jiaomen North Street, Beijing, 100068 China; 2 Department of Epidemiology and Health Statistics, School of Public Health, Capital Medical University, NO.10 Xitoutiao, Youanmen, Fengtai District, Beijing, 100069 China; 3 Department of Critical Care Medicine, Xuan Wu Hospital, Capital Medical University, no. 45 Changchun Street, Xicheng District, Beijing, 100053 China; 4 Medical Intensive Care Unit, Peking Union Medical College Hospital, Peking Union Medical College and Chinese Academy of Medical Sciences, NO.1 Shuaifuyuan, Dongcheng District, Beijing, 100730 China; 5 Department of Critical Care Medicine, Fu Xing Hospital, Capital Medical University, no. 20 Fuxingmenwai Street, Xicheng District, Beijing, 100038 China

**Keywords:** The modified nutrition risk in critically ill score, Intensive care unit, Mortality

## Abstract

**Background:**

Malnutrition in intensive care unit (ICU) patients is associated with adverse clinical outcomes. The modified nutrition risk in the critically ill score (mNUTRIC) was proposed as an appropriate nutritional assessment tool in critically ill patients, but it has not been fully demonstrated and widely used. Our study was conducted to identify the nutritional risk in ICU patients using the mNUTRIC score and explore the relationship between 28-day mortality and high mNUTRIC scores.

**Methods:**

This study is a secondary analysis, the data were extracted from The Beijing Acute Kidney Injury Trial (BAKIT). In total, 9049 patients were admitted consecutively, and 3107 patients with complete clinical data were included in this study. We divided the study population into high nutritional risk (mNUTRIC score ≥ 5 points) and low nutritional risk (mNUTRIC score < 5 points) groups. The predictive capacity of the mNUTRIC score was studied by receiver operating characteristic (ROC) curve analysis, appropriate cut-off was identified by highest combined sensitivity and specificity using Youden’s index. The significance level was set at 5%.

**Results:**

Among the 3107 patients, the 28-day mortality rate was 17.4% (540 patients died). Nearly 28.2% of patients admitted to the ICU were at risk of malnutrition, high nutritional risk patients were older (P < 0.001), with higher illness severity scores than low nutritional risk patients. Multivariate analysis revealed that the mNUTRIC score was an independent risk factor for 28-day mortality and mortality increased with increasing scores (p = 0.000). The calculated area under curve (AUC) for the mNUTRIC score was 0.763 (CI 0.740–0.786). According to Youden’s index, we found a suitable cut-off > 4 for the mNUTRIC score to predict the 28-day mortality.

**Conclusions:**

Patients admitted to the ICU were at high risk of malnutrition, and a high mNUTRIC score was associated with increased ICU length of stay and higher mortality. More large prospective studies are needed to demonstrate the validity of this score.

**Trial registration:**

This study was registered at www.chictr.org.cn (registration number Chi CTR-ONC-11001875). Registered on 14 December 2011.

**Supplementary Information:**

The online version contains supplementary material available at 10.1186/s12871-021-01439-x.

## Background

Malnutrition is common in intensive care unit (ICU) patients, it is associated with a variety of adverse outcomes, including higher complication rates, prolonged mechanical ventilation, prolonged hospitalization, and higher mortality [1, 2]. For critically ill patients, we should evaluate their nutritional status and provide adequate nutritional support [3], so effective tools are needed to assess the nutritional risk of ICU patients. However, traditional methods of nutrition assessment are limited in the hospital setting. Recently, Heyland et al. [4] published the first nutritional risk assessment tool specifically designed for critically ill patients: the NUTRIC score.

The NUTRIC score includes age, the Acute Physiology and Chronic Health Evaluation II (APACHE II) score [5], the Sequential Organ Failure Assessment (SOFA) score [6], comorbidities, days from hospitalization to ICU admission, and the interleukin-6 (IL-6) level, which was developed to link starvation, inflammation, and clinical outcomes. Patients are scored from 0 to 10, a score of 6 or greater indicates a high nutritional risk [4].

The NUTRIC score can predict 28-day mortality in a medical-surgical ICU population [4] and in postoperative surgical patients [7]. But the use of original NUTRIC score is limited by the availability of IL-6, which is not readily available in many institutions, and Heyland et al. stated that IL-6 only increased the C-index by 0.007 (from 0.776 to 0.783), with no statistical difference. Therefore, they suggested that in settings in which IL-6 is not available, it could be omitted from the NUTRIC score [4]. This adjusted score is called the modified NUTRIC score (mNUTRIC) [8]. Rahman et al. evaluated this modified NUTRIC score and found that mortality increased by 1.4% (95% CI, 1.3–1.5) for every point increase in the mNUTRIC score [8].

Some studies are available on the validity of the mNUTRIC score, however, most of them are small samples [9–12] or retrospective studies [13–15], and there are few prospective studies with large samples at present [8, 16, 17]. The mNUTRIC score has not been widely used in China, where has been no large sample studies. Moreover, there is a debate about the cutoff value of the mNUTRIC score [13, 14, 18]. Our main objective was to validate the mNUTRIC score in a surgical-medical ICU population in China, we also aimed to identify the cut-off point obtained in the mNUTRIC score that presented the best validity parameters for predicting mortality in this population.

## Methods

### Study design and data collection

**Fig. 1:**
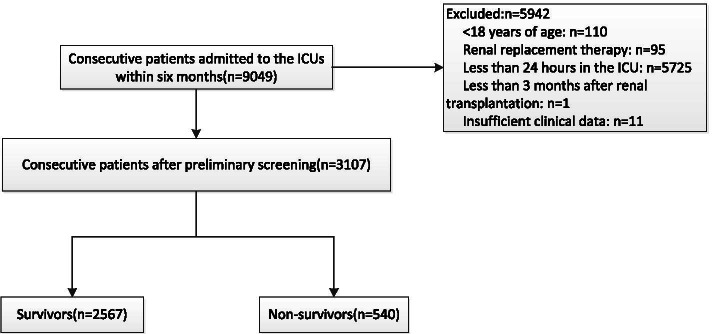
Study flowchart with 28-day mortality

This study used a database from a prospective, multi-centre, observational study that investigated the epidemiology of acute kidney injury (AKI) in critically ill patients in 30 ICUs at 28 tertiary hospitals in Beijing, China, from March 1 to August 31, 2012 (the Beijing Acute Kidney Injury Trial (BAKIT) [19] (for a complete list of these hospitals and the persons responsible for the data acquisition, see Additional file 1). Study subjects included all adult patients (age ≥ 18 years) admitted consecutively to the ICU. Only the initial ICU admission was considered in this study. The following patients were excluded: patients with preexisting end-stage chronic kidney disease, patients already receiving renal replacement therapy (RRT) before admission to the ICU, and patients who had received kidney transplantation in the previous 3 months [20]. Pre-existing comorbidities were diagnosed based on the International Classification of Diseases (ICD-10) codes. Patients were followed up until death, until hospital discharge, or for 28 days. Among the 9079 patients who were admitted consecutively, 3107 patients were included in our study (Fig. 1).

Thorough follow-up of all patients included in the study was conducted in the first 10 days after ICU admission. The collected data included demographics, anthropometrics, admission diagnosis, comorbidities, daily vital signs and laboratory data, which were used to automatically calculate the APACHE II score, the Simplified Acute Physiology Score II (SAPS II) score [21] and the SOFA score, days from hospital to ICU admission, ICU length of stay (LOS), hospital LOS, use of vasoactive drugs, and length of mechanical ventilation. RRT data were also reported.

Mortality data were collected up to 28 days after ICU discharge from hospital records, including records from hospital admissions and visits to outpatient clinics.

### Outcomes

The primary outcome was 28-day mortality, and the secondary outcome was the occurrence of the AKI.

### Nutritional support

Nutritional support methods were based on the guidelines for enteral and parenteral nutrition issued by the European and American Society of Enteroprotective Nutrition [22], combined with our accumulated clinical experience, individualized nutritional support was given to all patients. The patients began enteral nutrition (EN) 20–25 kcal/(kg.d) and a protein requirement of 1.2–2.0 g/(kg.d) within 24–48 h of admission to the ICU (on average). If the patient was intolerant of EN or had contraindications to EN, parenteral nutrition (PN) support was given within 24—48 h. If EN could not fully meet the nutritional needs of patients, appropriate intravenous supplementation with glucose, amino acids, or fat emulsion was given, that is, the combination of EN and PN.

### Definitions

We used the modified 9-point scale of the NUTRIC score, the mNUTRIC score [8]. We defined the scores from 0 to 4 as “low scores”, which indicated a low level of risk of malnutrition, and the scores from 5 to 9 as “high scores”, which were associated with worse clinical outcomes [8]. Because the mNUTRIC score includes APACHE II score, it was calculated only once at ICU admission.

AKI severity was classified according to the KDIGO guidelines [23]. AKI occurring within 10 days is defined as AKI, and more than 10 days is defined as non-AKI.

### Statistical analysis

Non-normally distributed continuous variables were expressed as the medians with interquartile ranges (IQRs) and were compared using the Mann–Whitney U test or Kruskal–Wallis analysis of variance with Bonferroni correction. Categorical variables were expressed as the number of cases and proportions and were compared using the Mantel–Haenszel Chi-square test.

A multivariate Cox regression analysis was performed using a backward stepwise selection method, with P value < 0.05 as the entry criterion, and P value ≥ 0.10 as the removal criterion. The assumption of proportional hazards was checked graphically using log (-log (survival probability)) plots and was found to be appropriate. Because the mNUTRIC score includes APACHE II and SOFA score, to avoid the duplicates, we did a collinearity analysis on the mNUTRIC score, APACHEII and SOFA, and found that there was no collinearity between them. Variables considered for multivariable analysis included age, sex, body mass index (BMI), APACHE II score, SAPS II, SOFA score, mNUTRIC score, use of vasoactive drugs, mechanical ventilation, AKI, RRT and underlying diseases. We tested for collinearity among all variables using a Cox regression analysis to generate hazard ratios (HR) and 95% confidence intervals (CIs).

The receiver operating characteristic (ROC) curve was drawn according to the sensitivity and specificity of the mNUTRIC score in predicting the 28-day mortality risk of patients and the best cut-off value was determined by the maximum of the Youden index (i.e., sensitivity plus specificity minus one) calculated from the ROC analysis. Using Hosmer- lemeshow goodness of fit to test the calibration of the scoring system. The 28-day survival stratified by low and high mNUTRIC scores was additionally evaluated graphically using the Kaplan–Meier product limit survival plot, we used Log-rank (Mantel–Cox) test for the comparison of survival curves. To verify the predictive effect of the mNUTRIC score on 28-day mortality in different populations, subgroup analysis was performed, we divided the study population into mechanical ventilation, medical mechanical ventilation (Because surgical patients are intubated for surgery, unlike medical patients who are intubated for serious medical conditions, we separately list medical patients who require intubation), sepsis, AKI and RRT patients, respectively.

All statistical analyses were performed using SPSS software (IBM Corp., Statistics for Windows, version 22.0, Armonk, NY, USA), with a two-sided P value < 0.05 considered statistically significant.

## Results

### Study population

Among the 9049 patients enrolled in the BAKIT study, 5942 were excluded for the reasons shown in Fig. 1, leaving 3107 patients for analysis. The characteristics of the entire cohort are shown in Table 1. The median age was 64 (IQR: 51–77) years, and 61.5% were men. The all-cause 28-day mortality rate was 17.4% and the median ICU LOS was 4 (IQR: 2–9) days. Among the included patients, the median BMI was 24 (IQR: 21–26) kg/m^2^, the median APACHE II score was 14 (IQR:10–20), the median SAPSII was 34 (IQR: 26–45), the median SOFA score was 6 (IQR: 3- 8), the median mNUTRIC score was 3 (IQR: 2–5), and the median number of comorbidities was 1 (IQR: 0—2). Mechanical ventilation was used in 2021 (65.0%) patients, 1307 patients (42.1%) received vasopressors, 1584 patients developed AKI and 281 patients (9.0%) underwent RRT. A total of 876 patients (28.2%) had high mNUTRIC scores.

**Table 1:** Patient characteristics by mNUTRIC score

Characteristic	All patients (n = 3107) Median(IQR) Number (%)	Low nutrition risk(mNUTRIC score ≤ 4, n = 2231) Median(IQR) Number (%)	High nutrition risk(mNUTRIC score ≥ 5, n = 876) Median(IQR) Number (%)	*P* value
Age(years)	64(51 -77)	60(47 -72)	76(66–82)	< 0.001
Male sex	1912(61.5)	1378(61.8)	534(61.0)	0.919
BMI(kg/m^2^)	24(21–26)	24(22–26)	23(21- 26)	0.003
Vasoactive therapy	1307(42.1)	954(42.8)	353(40.3)	0.457
Mechanical ventilation	2021(65.0)	1354(60.7)	667(76.1)	< 0.001
Sepsis	896(28.8)	419(18.8)	477(54.5)	< 0.001
RRT	281(9.0)	108(4.8)	173(19.7)	< 0.001
**Severity of illness**				
APACHEII	14(10–20)	12(8–15)	23(19- 28)	< 0.001
SAPSII	34(26–45)	30(23–38)	50(39- 64)	< 0.001
SOFA	6(3–8)	4(3–7)	9(6–11)	< 0.001
mNUTRIC score	3(2–5)	3(2–3)	6(5–7)	< 0.001
**Admission category**				
medical	1480(47.6)	878(39.4)	602(68.7)	< 0.001
surgical	1627(52.4)	1353(60.6)	274(31.3)	
**Comorbid diseases**				
Cancer	486(15.6)	297(13.3)	189(21.6)	
Hypertension	1222(39.3)	739(33.1)	483(55.1)	
Coronary disease	615(19.8)	293(13.1)	322(36.8)	
Chronic kidney disease	170(5.5)	63(2.8)	107(12.2)	
Diabetes	532(17.1)	277(12.4)	255(29.1)	
COPD	166(5.3)	89(4.0)	77(8.8)	
**Category of ICU admission diagnosis**				
Cardiovascular	848(27.3)	681(30.5)	167(19.1)	
Respiratory	548(17.6)	316(14.2)	232(26.5)	
Neurologic	462(14.9)	321(14.4)	141(16.1)	
Trauma	238(7.7)	191(8.6)	47(5.4)	
Gastrointestinal	607(19.4)	413(18.5)	194(22.1)	
Metabolic	77(2.5)	43(1.9)	34(3.9)	
**Outcome data**				
ICU LOS(days)	4(2–9)	4(2–7)	6(3–13)	< 0.001
Hospital LOS(days)	19(12–29)	19(12–28)	21(11–34)	0.002
28-day mortality	540(17.4)	208(9.3)	332(37.9)	< 0.001
In-hospital mortality	521(16.8)	173(7.8)	348(39.7)	< 0.001
AKI	1584(51.0)	929(41.6)	655(74.8)	< 0.001
Hospitalization expense(thousand yuan)	40(19–96)	34(17–87)	55(27–113)	< 0.001

Data are expressed as the median (interquartile range), and number (percentage). BMI, body mass index; SAPS II, Simplified Acute Physiology Score II; SOFA, Sequential Organ Failure Assessment; APACHE II, Acute Physiology and Chronic Health Evaluation II; mNUTRIC score, the modified nutrition risk in the critically ill score; COPD, chronic obstructive pulmonary disease; LOS, length of stay; AKI, acute kidney injury; RRT, renal replacement therapy

### Characteristics of high nutritional risk patients

From Table 1, we can see high nutritional risk patients were older (P < 0.001), with higher illness severity scores than low nutritional risk patients. High nutritional risk patients were more likely to present with sepsis on ICU admission, were more likely to develop AKI, and had longer durations of ICU and hospital stays when compared to the low nutritional risk group. Furthermore, mechanical ventilation was more commonly used in high nutritional risk patients (76.1% vs 60.7%; P < 0.001). The 28-day mortality and in-hospital mortality rates were higher among high nutritional risk patients than low nutritional risk patients (P < 0.001).

### 28-Day mortality according to score

**Fig. 2:**
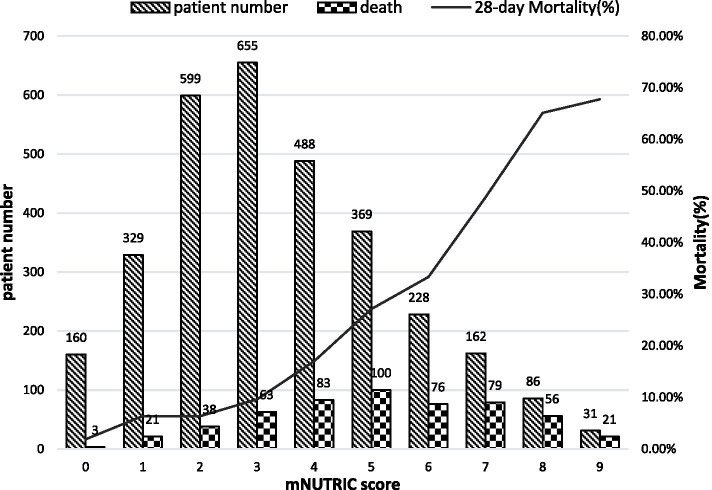
The 28-day mortality according to mNUTRIC score

Our analysis showed that the 28-day mortality increased with higher mNUTRIC scores (Fig. 2), and the 28-day mortality for the maximum mNUTRIC score was 67.4%.

### High mNUTRIC score and the 28-day mortality

**Fig. 3:**
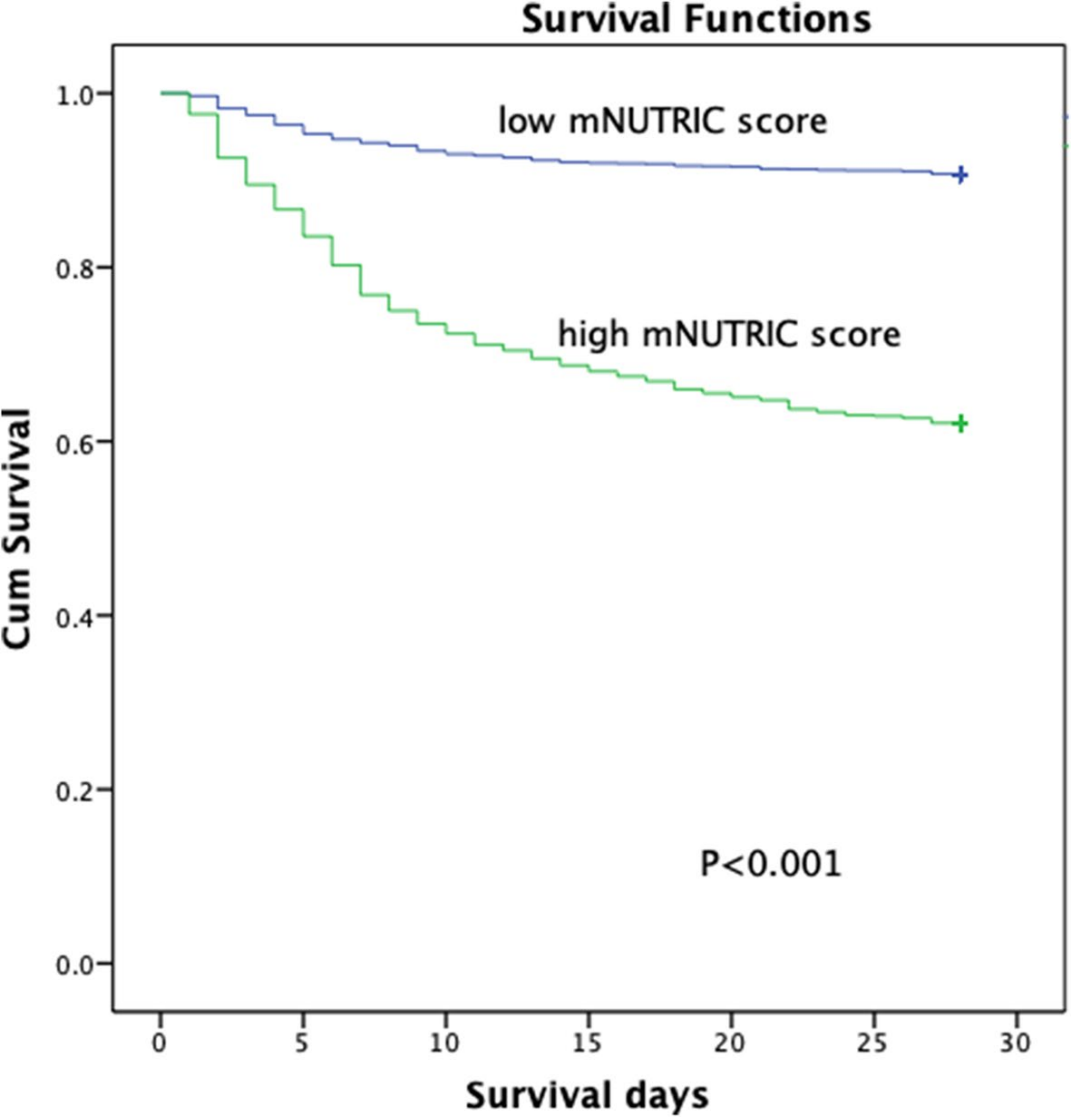
Survival curve of 28-day mortality stratified by mNUTRIC scores

In multivariate Cox regression analysis (Table 2), after adjusting for age, sex, BMI, sepsis, APACHE II score, SAPS II, SOFA score, mNUTRIC score, use of vasoactive drugs, mechanical ventilation, AKI, RRT and underlying diseases, the mNUTRIC score, APACHE II score, SAPS II, sepsis, mechanical ventilation, AKI and RRT were independent predictors of 28-day mortality, and the 28-day mortality increased by 8.5% for every point increase in the mNUTRIC score (p = 0.012, HR = 1.085). Kaplan–Meier analysis also showed that the presence of high mNUTRIC scores was associated with a higher risk of mortality (p < 0.001) (Fig. 3).

**Table 2:** Multivariate Cox regression analysis of 28-day mortality in all patients

Characteristic	Hazard ratio	95%CI	P
mNUTRIC score	1.085	1.019–1.156	0.011
APACHEII	1.025	1.006–1.045	0.010
SAPSII	1.022	1.014–1.030	0.000
Sepsis	1.721	1.407–2.104	0.000
MV	1.533	1.031–2.279	0.035
AKI	2.148	1.703–2.710	0.000
RRT	1.296	1.043–1.610	0.019

mNUTRIC score, the modified nutrition risk in the critically ill score; APACHE II, Acute Physiology and Chronic Health Evaluation II; SAPS II, Simplified Acute Physiology Score II; MV, mechanical ventilation; AKI, acute kidney injury; RRT, renal replacement therapy; HR, hazard ratio; CI, confidence interval

### Area under the curve of scores for predicting 28-day mortality

**Fig. 4:**
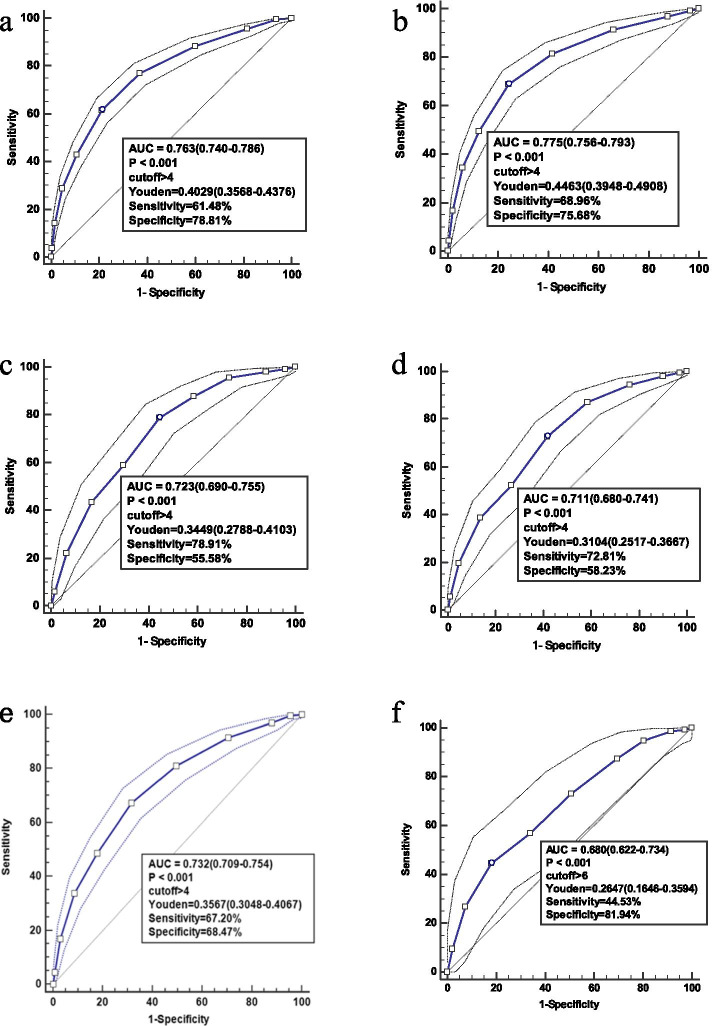
Performance of mNUTRIC scores in predicting 28-day mortality. **a**. All patients (n = 3107); **b**. All mechanical ventilation patients (n = 2021); **c**. Medical mechanical ventilation patients (n = 751); **d**. Sepsis patients (n = 896); **e**. AKI patients (n = 1584); f. RRT patients (n = 281)

We divided the study population into mechanical ventilation, medical mechanical ventilation, sepsis, AKI and RRT patients, respectively. We can see that in this cohort and each subgroup, the areas under the curve (AUCs) of the mNUTRIC score for predicting 28-day mortality indicated good predictive performance of the score (Fig. 4). In the ROC curve for the mNUTRIC score, the best cut-off value was at 4 (sensitivity 61.48% and specificity 78.81%) in this cohort, and the Youden index was 0.4029.

## Discussion

This study was a secondary analysis of a prospective observational study in surgical-medical ICUs. We used a validated nutrition assessment tool in an attempt to demonstrate an association between malnutrition and 28-day mortality. We found a high incidence of malnutrition in ICU patients, and malnutrition was associated with a poor prognosis.

In the present study, 28.2% of the critically ill patients admitted to the ICU were at high nutritional risk (mNUTRIC scores ≥ 5). These findings were similar to the results of a study conducted in Turkey [7], in which 22.4% patients were evaluated as having high scores (between 5 and 9). Lew et al. [24] also demonstrated that the prevalence of malnutrition in the ICU was 28% using the 7-point Subjective Global Assessment (7-point SGA) to determine patients’ nutritional status. Recently, a study [10] reported that 45% of mechanically ventilated patients admitted to the ICU were at high nutritional risk. Similarly, Kalaiselvan et al. [25] reported that 42.5% of mechanically ventilated patients had NUTRIC scores ≥ 5. Our study is more generalizable because of the inclusion of both medical and surgical patients. The aforementioned studies included only patients on mechanical ventilation, and patients on mechanical ventilation were more seriously ill than those not on mechanical ventilation. The differences among studies are mainly the result of different populations and nutrition screening tools.

In our study, the 28-day mortality associated with the maximum mNUTRIC score was 67.7%, which is similar to the finding in the study by Jeong [13], in which this rate was 62.5%. Compared with patients with a low NUTRIC score, patients with high NUTRIC score had a higher mortality rate and longer ICU LOS, similar results were reported by other studies [4, 16].

The mortality rate in our study was 17.4%, which was lower than the rate reported in the second validation study of the NUTRIC score (29%) by Rahman et al. [8]. This difference may be because our study included many postoperative care patients. In this study, we found that the mNUTRIC score was a good prognostic predictor in critically ill patients and that high mNUTRIC scores were associated with an elevated risk of death at 28 days (HR = 1.085, 95% CI = 1.018 to 1.157, P = 0.012). This finding is consistent with those of prior studies [9, 13, 16].

The mNUTRIC score was found to have a fair predictive performance for 28-day mortality in this cohort (AUC 0.763; 95% CI 0.740—0.786) and each subgroup. These results are in line with those of the initial validation study by Heyland et al. (AUC: 0.783) [4] and a recently published validation study of the mNUTRIC score by Mukhopadhyay et al. (AUC 0.71) [26]. Recently, a study [13] showed that the AUC of the NUTRIC score for the prediction of 28-day mortality was 0.762 (95% CI: 0.718–0.806), while that of the mNUTRIC score was 0.757 (95% CI: 0.713–0.801). There was no significant difference between the two scores (p = 0.45). The mNUTRIC score is a good nutritional risk assessment tool for critically ill patients.

We found that the best cut-off value for the mNUTRIC score was > 4 (sensitivity 61.48% and specificity 78.81%) in this cohort, and the Youden index was 0.4029, which is consistent with previous work by de Vries et al. [14]. However, in another study, the best cut-off value was at 6 (sensitivity 75% and specificity 65%), and the Youden index was 0.401[13]. Jung et al. reported that patients were considered to be at high risk of malnutrition when their mNUTRIC score was ≥ 5[27]. Our study included patients with various diseases, while Jung’s study population was limited to patients with sepsis. Further investigation is needed to find the best cut-off value of the mNUTRIC score to define the high-risk group.

The limitations of our study stem mainly from the fact that it is a secondary analysis of an original database that lacked data on inflammation indicators such as IL-6. Therefore, we could not calculate the NUTRIC score to verify the difference between the NUTRIC and mNUTRIC score. Second, nutrition history and feeding parameters were not available in our cohort, so the associations among nutritional adequacy, mNUTRIC score and mortality could not be confirmed by our results. Third, we did not perform dynamic nutritional risk assessments, which may provide more information for patient outcomes.

## Conclusion

Patients were considered to be at high risk of malnutrition when their mNUTRIC score was > 4. The mNUTRIC score is a practical, easy-to-use tool based on variables that are easy to obtain in the critical care setting.

## Supplementary Information

**Additional file 1**

**Additional file 2**

## Data Availability

The datasets used and analyzed during the current study are available from the corresponding author on reasonable request. Corresponding author: Xiuming Xi, email: xixiuming2937@sina.com.

## Abbreviations

ICU: Intensive care unit
NUTRIC: the nutrition risk in the critically ill score
IL-6: Interleukin-6
mNUTRIC: The modified nutrition risk in the critically ill score
ROC: Receiver operating characteristic
AUC: Area under curve
AKI: Acute kidney injury
BAKIT: The Beijing Acute Kidney Injury Trial
BMI: Body mass index
RRT: Renal replacement therapy
APACHE II: Acute physiology and chronic health evaluation II
SAPS II: The simplified acute physiology score II
SOFA: Sequential organ failure assessment
LOS: Length of stay
EN: Enteral nutrition
PN: Parenteral nutrition
IQR: Interquartile range
HR: Hazard ratio
CI: Confidence interval
COPD: Chronic obstructive pulmonary disease
