# Supplementary material for: Association between the modified Nutrition Risk in Critically Ill (mNUTRIC) score and clinical outcomes in the intensive care unit: a secondary analysis of a large prospective observational study
Source: BMC Anesthesiol. 2021 Sep 8;21:220. doi: 10.1186/s12871-021-01439-x (PMC8424878; doi:10.1186/s12871-021-01439-x)
Supplement: Supplementary file 1 — Additional file 1 [file 12871_2021_1439_MOESM1_ESM.docx]

### *Additional file 1:*

**Members of the Beijing Acute Kidney Injury Trial (BAKIT) workgroup**

Yuan Xu (xyuan76@sohu.com), Department of Critical Care Medicine, Beijing Tongren Hospital, Capital Medical University, Beijing 100730, China

Jianxin Zhou (jianxinz@yeah.net), Department of Critical Care Medicine, Beijing Tiantan Hospital affiliated to Capital Medical University, Beijing 100050, China

Ang Li (liang_bjfh68@hotmail.com), Department of Critical Care Medicine, Beijing Friendship Hospital, Capital Medical University, Beijing 100050, China

Jingyuan Liu (liujingyuan.bj@hotmail.com), Department of Critical Care Medicine, Beijing Ditan Hospital , Capital Medical University, Beijing 100015, China

Wenxiong Li ([lwx7115@sina.com](mailto:lwx7115@sina.com)), Surgical Intensive Care Unit, Beijing Chaoyang Hospital , Capital Medical University, Beijing 100050, China

Wenjin Chen (drchenwenjin@gm ) Neurological intensive care unit, Xuanwu Hospital, Capital Medical University, Beijing 100053, China

Jianguo Jia (jiajianguo_1@126.com), Surgical Intensive Care Unit, Xuanwu Hospital, Capital Medical University, Beijing 100053, China

Xi Zhu (xizhuccm@163.com), Department of Critical Care Medicine, Peking University Third Hospital, Beijing 100191, China

Penglin Ma (mapenglin1@163.com), Department of Critical Care Medicine, The 309th Hospital of Chinese People’s Liberation Army, Beijing 100094, China

Wei Chen (hanwa@yahoo.com.cn) Department of Critical Care Medicine, Beijing Shijitan Hospital, Capital Medical University, Beijing 100038, China

Dongxin Wang (wangdongxin@hotmail.com), Department of Critical Care Medicine, Peking University First Hospital, Beijing 100034, China

Youzhong An (bjicu@163.com), Department of Critical Care Medicine, Peking University People’s Hospital, Beijing 100044, China

Qingyuan Zhan (Zhanqy0915@yahoo.com.cn), Department of Critical Care Medicine, China-Japan Friendship Hospital, Beijing 100029, China

Gang Li (xdysw@163.com), Department of Critical Care Medicine, China-Japan Friendship Hospital, Beijing 100029, China

Haitao Zhang ([boy398672@yahoo.cn](mailto:boy398672@yahoo.cn)), Surgical Intensive Care Unit, Fuwai Hospital, China Academy of Medical Science and Peking Union Medical College, Beijing 100037, China

Bo Ning ([ningboicu@tom.com](mailto:ningboicu@tom.com) ), Department of Critical Care Medicine, Air Force General Hospital of Chinese People’s Liberation Army, Beijing 100142, China

Zhongjie He ([drhezhj@126.com](mailto:drhezhj@126.com)), Department of Critical Care Medicine, The First Affiliated Hospital of General Hospital of People’s Liberation Army, Beijing 100048, China

Zhicheng Zhang ([zhangzhichengicu@hotmail.com](mailto:zhangzhichengicu@hotmail.com)), Department of Critical Care Medicine, Navy General Hospital, Beijing 100048, China

Yaxiong Sun ([1073791787@qq.com](mailto:1073791787@qq.com)), Department of Critical Care Medicine, The Luhe Teaching Hospital of the Capital Medical University, Beijing 101149, China

Shijie Jia ( jiashj1964@sina.com), Surgical Intensive Care Unit, Beijing Anzhen Hospital, Capital Medical University, Beijing 100029, China

Yalin Liu ([icu9999@sina.com.cn](mailto:icu9999@sina.com.cn)), Surgical Intensive Care Unit, Beijing Hospital, Beijing 100005, China

Rui Cheng ([chengrui2017@163.com](mailto:chengrui2017@163.com)), Department of Critical Care Medicine, General Hospital of Armed Police Forces, Beijing 100039, China

Qing Song (songqing3010301@sina.com), Department of Critical Care Medicine, The General Hospital of People’s Liberation Army, Beijing 100039, China

Jinning Liu (jin_ning_liu@163.com ), Surgical Intensive Care Unit, Beijing YouAn Hospital, Capital Medical University, Beijing 100069, China

Yangong Chao (chaoyg1059@263.net), Department of Critical Care Medicine, Hua Xin Hospital, First Hospital of Tsinghua University , Beijing 100016, China

Huizhen Li ([huizl630@163.com](mailto:huizl630@163.com)), Department of Critical Care Medicine, Beijing Shunyi Hospital of China Medical University, Beijing 101300, China

Li Feng (bjfengli668@sina.com), Department of Critical Care Medicine, Beijing Geriatric Hospital, Beijing 100095, China

Ruochun Shi (jinxier@163.com), Department of Critical Care Medicine, Beijing No.6 Hospital, Beijing 100007, China

Department of Critical Care Medicine, Fuxing Hospital, Capital Medical University, Beijing 100038, China: Ying Wen (Christina7622@163.com), Qi Jiang ([jiangqi7676@sina.com](mailto:jiangqi7676@sina.com)), Peng Wang ([438867228@qq.com](mailto:438867228@qq.com)), Yujie Deng ([missydyj@gmail.com](mailto:missydyj@gmail.com)), Yan Sun ([sunyan198408011717@163.com](mailto:sunyan198408011717@163.com)), Yanyan Yin ([yinyanyan678@163.com](mailto:yinyanyan678@163.com)), Xin Zhang ([wood678@sohu.com](mailto:wood678@sohu.com)), Li Zhang ([letmedo@sina.com](mailto:letmedo@sina.com)), Zhen Zhao ([maggiezhao77@163.com](mailto:maggiezhao77@163.com)), Ying Wang ([butterfly_5643358@hotmail.com](mailto:butterfly_5643358@hotmail.com)), Ran Lou ([springin12@sina.com](mailto:springin12@sina.com)), Jing Wang ([monica_jojo2003@yahoo.com.cn](mailto:monica_jojo2003@yahoo.com.cn))
